# Magnetoelastic Effect in Ni-Zn Ferrite Under Torque Operation

**DOI:** 10.3390/ma17246239

**Published:** 2024-12-20

**Authors:** Jacek Salach, Maciej Kachniarz, Dorota Jackiewicz, Adam Bieńkowski

**Affiliations:** Institute of Metrology and Biomedical Engineering, Warsaw University of Technology, A. Boboli 8, 02-525 Warsaw, Poland; jacek.salach@pw.edu.pl (J.S.); dorota.jackiewicz@pw.edu.pl (D.J.); adam.bienkowski@pw.edu.pl (A.B.)

**Keywords:** magnetoelastic effect, torque operation, soft magnetic material, Ni-Zn ferrite, magnetoelastic sensitivity

## Abstract

The magnetoelastic effect is known as the dependence between the magnetic properties of the material and applied mechanical stress. The stress might not be applied directly but rather generated by the applied torque. This creates the possibility of developing a torque-sensing device based on the magnetoelastic effect. In this paper, the concept of an axially twisted toroidal magnetic core as a torque-sensing element is considered. Most known works in this field consider the utilization of an amorphous ribbon as the core material. However, Ni-Zn ferrites, exhibiting relatively high magnetostriction, also seem to be promising materials for magnetoelastic torque sensors. This paper introduces a theoretical description of the magnetoelastic effect under torque operation on the basis of total free energy analysis. The methodology of torque application to the toroidal core, utilized previously for coiled cores of amorphous ribbons, was successfully adapted for the bulk ferrite core. For the first time, the influence of torque on the magnetic properties of Ni-Zn ferrite was investigated in a wide range of magnetizing fields. The obtained magnetoelastic characteristics allowed the specification of the magnetoelastic torque sensitivity of the material and the determination of the optimal amplitude of the magnetizing field to maximize this parameter. High sensitivity, in comparison with previously studied amorphous alloys, and monotonic magnetoelastic characteristics indicate that the investigated Ni-Zn ferrite can be utilized in magnetoelastic torque sensors. As such, it can be used in torque-sensing applications required in mechanical engineering or civil engineering, like the evaluation of structural elements exposed to torsion.

## 1. Introduction

The manufacturing and utilization of soft magnetic materials are among the more important aspects of modern industrial activity. The value of the global market of soft magnetic materials is estimated to be USD 34.1 billion in the year 2024 [1]. For the years 2025–2033, constant growth in the market is expected, which is indicated by the predicted compound annual growth rate (CAGR) of 7% [1]. Among the many functional features of soft magnetic materials, magnetomechanical properties are particularly interesting. A change in the magnetic state of the material during the operation of force or torque creates the possibility of applying soft magnetic materials in mechanical sensors.

Torque measurement plays an important role in modern mechanical engineering. It is required, for example, in rotational movement transmission systems, the measurement of friction in bearings and the evaluation of structural elements exposed to torsion. Measuring torque is also crucial for the operation of electronic and programmable torque wrenches.

The most commonly utilized type of torque sensor is the strain gauge [2]. However, magnetoelastic torque sensors are also a dynamically advancing group. The principle of operation is based on a change in the magnetic properties of the ferromagnetic material under the influence of shear stress resulting from applied torque. There are several solutions in the field of magnetoelastic torque sensors in the literature. These mainly include cross-shaped heads for contactless torque measurement in ferromagnetic shafts (torductor) [3,4,5], sensors utilizing magnetostrictive strips (particularly those made of thin magnetic ribbons) rigidly mounted (glued or pressed) on the surface of a twisted shaft [6,7,8,9,10,11,12] and sensors based on twisted amorphous wires utilizing the Inverse Wiedemann effect or the Matteucci effect [13,14,15,16]. The first two methods require an additional setup for the measurement of the magnetic parameters of the twisted magnetic shaft or magnetostrictive strips mounted on the nonmagnetic shaft. This increases the complexity of the solution. In the case of sensors based on magnetic strips, there is also the risk of introducing the initial stress while rigidly mounting them on the surface of the shaft. Moreover, in all three cases, the material forms an open magnetic circuit, and demagnetization energy has to be taken into consideration, which limits the magnetoelastic sensitivity. The possible solution to these drawbacks is the utilization of a toroidal magnetic core twisted along the axis, which was discussed previously in [17,18]. Such a solution provides a closed magnetic circuit of the core and allows for obtaining a uniform distribution of shear stress along the entire magnetic flux flow path. The toroidal core is both a torque-transmitting element and a measurement transducer at the same time. Thus, there is no requirement for a rigid connection with the shaft, reducing the risk that the initial stress affects the measurement results.

In terms of magnetic material for the toroidal core of the torque sensor, ferromagnetic Fe-based amorphous and nanocrystalline alloys magnetized in relatively strong magnetic fields (near saturation) have been considered so far. The toroidal core is is coiled of thin ribbon. Such cores exhibited high magnetoelastic torque sensitivity, but mostly after additional thermal processing [17,18]. Similar behavior was observed in terms of axial tensile or compressive stress when higher sensitivity was obtained after annealing [19]. The high magnetoelastic sensitivity of Fe-based amorphous alloys is attributed to the significant magnetostriction coefficient. However, reaching the maximum sensitivity requires thermal annealing, reducing the internal stress induced in the rapid quenching manufacturing process [20,21,22]. On the other hand, some nickel–zinc (Ni-Zn) ferrites are also known to exhibit significant magnetostriction, albeit to a lesser degree than Fe-based amorphous alloys [23]. It was previously proven that these materials are also highly sensitive to axial stress [24]; however, reaching full sensitivity does not require additional thermal processing. Moreover, recent research indicated that lowering the amplitude of the magnetizing field can positively affect the stress sensitivity, changing the relation between magnetoelastic energy and magnetizing field energy [25]. Therefore, it can be assumed that Ni-Zn ferrite can also exhibit significant torque (shear stress) sensitivity when formed into a toroidal core subjected to axial torque. Yet, such an application of Ni-Zn ferrite was not previously considered.

As a material for the toroidal core of a magnetoelastic torque sensor, ferrites seem to be more favorable than amorphous alloys. Rapidly quenched amorphous materials are manufactured mostly in the form of planar structures: foils and ribbons. Therefore, the toroidal core has to be formed from the ribbon, which additionally increases the complexity of the core preparation. Ferrite, on the other hand, can be formed into the desired shape during the manufacturing process and does not require additional procedures while preparing the core. It also provides greater flexibility in terms of the core shape and dimensions. However, ferrites are generally brittle, which results in poor performance under tensile stress. As the shear stress introduced by the torque can be decomposed into tensile and compressive stress, ferrites may exhibit lower shear strength than more elastic amorphous alloys. This might limit the range of torque that can be measured without damaging the core. Yet, it has to be noted that after thermal annealing, when amorphous alloys reach their maximum torque sensitivity, their brittleness also increases, which may reduce the range of torque operation.

The aim of the following paper is to investigate the influence of torque on the magnetic characteristics of a selected Ni-Zn ferrite in a wide range of magnetizing fields and to determine the optimal magnetization conditions for torque-sensing applications. The obtained torque sensitivity will also be compared with previous results for amorphous alloys.

## 2. Magnetoelastic Effect Under Torque Operation

The classic magnetoelastic effect, known as the Villari effect, involves change in the magnetic state of the ferromagnetic or ferrimagnetic material under the influence of external uniaxial stress, tensile or compressive [26]. As that, it is a phenomenon that is inverse to the magnetostriction. The change in magnetic state of the stressed material can be observed as the variation of the magnetization *M* or magnetic flux density *B* in the set magnetizing field *H*. Therefore, the influence of stress σ on the magnetic material is often represented as parametric magnetoelastic characteristics $B_{m}{\left(\sigma \right)}_{H_{m}}$, where $H_{m}$ and $B_{m}$ are the maximum values of the magnetizing field and magnetic flux density, respectively [19,24,27,28]. The effect of stress can be observed in the wide range of magnetizing fields; however, the scale of changes in $B_{m}$ under stress depends on the $H_{m}$ value [25].

The magnetoelastic effect is usually considered on the basis of the total free energy of the magnetic material. When referring to the material in general and not a specific sample, it is convenient to use the concept of energy density *w* expressed in J/m^3^. Therefore, the total free energy density $w_{T}$ of the magnetic material can be expressed as [29,30]:

(1) $$w_{T}=w_{E}+w_{A}+w_{D}+w_{H}+w_{\sigma }$$

where $w_{E}$ is the exchange interaction energy density, $w_{A}$ is the magnetocrystalline anisotropy, $w_{D}$ is the shape anisotropy (demagnetization energy density), $w_{H}$ is the magnetizing filed energy density (Zeeman energy density) and $w_{\sigma }$ is the magnetoelastic anisotropy. The minimum of the total free energy density $w_{T}$ determines the orientation of the spontaneous magnetization $M_{s}$ vector of the magnetic domain. In a simplified approach, the main components influencing the magnetic state of the material are energy densities resulting from the external sources: magnetizing field *H* and uniaxial stress σ. The energy density of the magnetizing field *H* (Zeeman energy density) can be expressed as [26,27]:

(2) $$w_{H}=-\mu_{0}HM_{s}\cos\left(\psi -\varphi \right)$$

where $\mu_{0}=4\pi \times 10^{-7}$ H/m is the magnetic permeability of free space, ψ is the angle between magnetizing field *H* and the magnetic anisotropy axis and φ is the angle between magnetization $M_{s}$ and the anisotropy axis. The magnetoelastic energy density (magnetoelastic anisotropy), with the assumption of isotropic magnetostriction of the material reducing the problem to the uniaxial anisotropy, is given as [26,29,30,31]

(3) $$w_{\sigma }=\frac{3}{2}\lambda_{s}\sigma \sin^{2}\varphi$$

where $\lambda_{s}$ is the saturation magnetostriction coefficient and φ is again the angle between magnetization $M_{s}$ and the axis of the anisotropy introduced by stress σ—the magnetoelastic anisotropy. In the simplified case where only Zeeman energy density $w_{H}$ and magnetoelastic anisotropy energy density $w_{\sigma }$ are considered, the free energy density of the system can be expressed as

(4) $$w=w_{H}+w_{\sigma }=-\mu_{0}HM_{s}\cos\left(\psi -\varphi \right)+\frac{3}{2}\lambda_{s}\sigma \sin^{2}\varphi$$

where ψ is now the angle between magnetizing field *H* and the direction of the applied stress σ (magnetoelastic anisotropy axis). Most of the considerations in the literature were carried out under the assumption of a parallel introduction of magnetizing field *H* and uniaxial stress σ, simplifying Equation (4) to the following form [32,33]:

(5) $$w=-\mu_{0}HM_{s}\cos\varphi +\frac{3}{2}\lambda_{s}\sigma \sin^{2}\varphi$$

**Figure 1:**
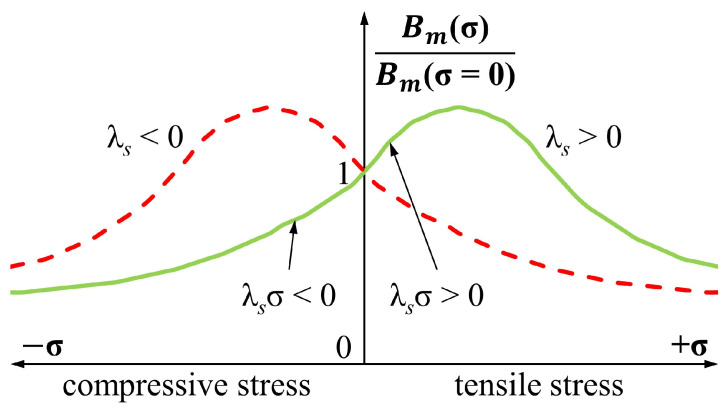
Influence of the axial stress σ on the normalized maximum magnetic flux density $B_{m}$ for the material with positive ($\lambda_{s}>0$, solid green line) and negative ($\lambda_{s}<0$, dashed red line) magnetostriction [25,28,31].

While Zeeman energy density forces the magnetization $M_{s}$ to direction φ=0 (energy minimum) parallel to the magnetizing field *H*, the influence of the magnetoelastic anisotropy depends on the sign of the $\lambda_{s}\sigma$ factor. However, due to the twofold symmetry of the magnetoelastic anisotropy (sin^2^ *φ*), there are two equal energy minima. Therefore, for $\lambda_{s}\sigma >0$, stress σ forces the magnetization $M_{s}$ into a direction parallel φ=0 or antiparallel φ=π, while $\lambda_{s}\sigma <0$ results in magnetization $M_{s}$ tending to orient along perpendicular direction $\varphi =\frac{\pi }{2}$ or $\varphi =\frac{3\pi }{2}$ [29]. Thus, it can be seen that the interaction of both energy densities may have a cooperative ($w_{H}$ and $w_{\sigma }$ forces $M_{s}$ into the same direction) or competitive ($w_{H}$ and $w_{\sigma }$ forces $M_{s}$ into different directions) nature. This gives rise to the typical magnetoelastic $B_{m}{\left(\sigma \right)}_{H_{m}}$ characteristics presented in Figure 1, where either an increase or decrease in $B_{m}$ with a stress value is possible depending on the said nature of the energy sources’ interaction. As the macroscopic magnetization *M* and therefore the magnetic flux density *B* are governed by the energy supplied with both magnetizing field *H* and stress σ, the resulting magnetic state of the material is mostly influenced by the stronger energy source. Thus, it can be expected that for lower magnetizing fields, the influence of stress is more significant, leading to higher magnetoelastic sensitivity. This assumption was confirmed for uniaxial stress in previous research [25,34].

**Figure 2:**
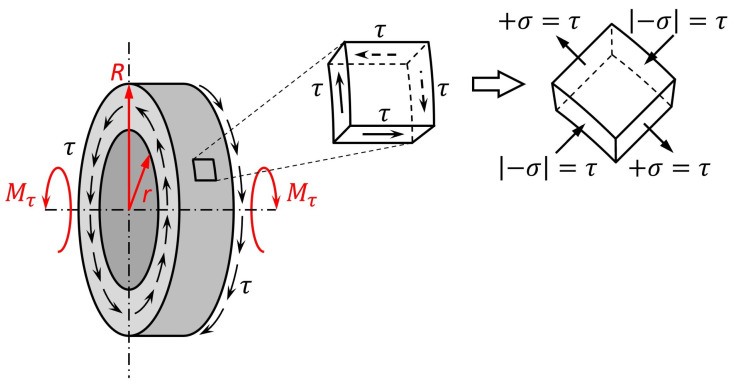
Schematic representation of the stress state of toroidal core subjected to the axial torque $M_{\tau }$. For the pure torsion, shear stress τ in elementary excerpt can be decomposed into the pair of principal stress of value equal to τ: +σ=|−σ|=τ [31].

The magnetoelastic effect is also observed under shear stress resulting from the applied torque. In such conditions, the character of the relationship between the magnetic state and introduced stress is however more complex and depends on the core geometry. The stress state of the toroidal core subjected to axial torque $M_{\tau }$ is schematically presented in Figure 2. The shear stress τ introduced by torque $M_{\tau }$ depends on the distance ρ from the toroid axis:

(6) $$\tau \left(\rho \right)=\frac{M_{\tau }\rho }{J_{0}}$$

where $J_{0}$ is the torsional constant expressed for toroid as

(7) $$J_{0}=\frac{\pi }{2}\left(R^{4}-r^{4}\right)$$

*R* and *r* being the outer and inner radius, respectively. Under the pure torsion, the shear stress τ in the elementary excerpt of the toroid can be decomposed into a pair of the principal stress (Figure 2) values equal to τ. Therefore, the acting tensile and compressive stress can be expressed as

(8) $$+\sigma =\left|-\sigma \right|=\tau =\frac{2M_{\tau }\rho }{\pi \left(R^{4}-r^{4}\right)}$$

As the toroidal core is magnetized along the perimeter, the distribution of stress is uniform along the magnetic flux flow path; however, it varies with ρ along the cross-section of the magnetic circuit. Both +σ and −σ stress create a common anisotropy axis (if $\lambda_{s}\sigma >0$, the anisotropy axis is parallel to the +σ direction and perpendicular to the −σ direction, opposite to $\lambda_{s}\sigma <0$) at $\frac{\pi }{4}$ angle against the magnetic field *H* direction [31,35]. Therefore, Equation (4) can be rewritten as [35]

(9) $$w=-\mu_{0}HM_{s}\cos\left(\frac{\pi }{4}-\varphi \right)+\frac{3}{2}\lambda_{s}\sigma \sin^{2}\varphi -\frac{3}{2}\lambda_{s}\sigma \sin^{2}\left(\varphi +\frac{\pi }{2}\right)$$

Substituting (8) and reducing the equation leads to

(10) $$w=-\mu_{0}HM_{s}\cos\left(\frac{\pi }{4}-\varphi \right)-\frac{3\lambda_{s}M_{\tau }\rho }{\pi \left(R^{4}-r^{4}\right)}\cos2\varphi$$

Taking the anisotropy axis as the reference direction, analysis of the components of the equation shows that Zeeman energy density $w_{H}$ forces the vector of magnetization $M_{s}$ into $\varphi =\frac{\pi }{4}$ direction, for which the $w_{H}$ component reaches its minimum. Simultaneously, the magnetoelastic anisotropy $w_{\sigma }$ tends to orient the magnetization $M_{s}$ along the anisotropy axis, when 2φ=0 or 2φ=2π and $w_{\sigma }$ reaches minimum. The preferred $M_{s}$ orientation for $w_{\sigma }$ minimum is therefore either parallel (φ=0) or antiparallel (φ=π) to the anisotropy axis. As both orientations are equally probable, torsion alone is not able to magnetize the material and total magnetization remains close to 0 when the magnetizing field is absent (H=0). However, in the presence of magnetizing field *H*, the applied torque influences total magnetization and the magnetic state of the axially twisted toroidal core is again the result of interaction between the Zeeman energy density $w_{H}$ and the magnetoelastic anisotropy energy density $w_{\sigma }$. Yet due to the non-parallel direction corresponding to each energy density minimum, the nature of their interaction is always competitive, unlike in the case of the classic Villari effect. Therefore, the $B_{m}{\left(M_{\tau }\right)}_{H_{m}}$ characteristic exhibits a monotonic decrease of $B_{m}$ with a $M_{\tau }$ value unlike the characteristic for axial stress presented in Figure 1.

As the magnetic state of the material is shaped by the competition between Zeeman energy density $w_{H}$ and magnetoelastic anisotropy energy density $w_{\sigma }$, their ratio is essential for the torque sensitivity of the material. Similarly to the case of axial stress, it can thus be expected that reducing the amplitude of magnetizing field $H_{m}$ can positively affect the sensitivity. Therefore, the investigation of torque on the magnetic state of Ni-Zn ferrite was performed for the wide range of magnetizing fields, from the low magnetizing field region (Rayleigh region) to the magnetic saturation region. This allowed to verify the discussed theoretical description and determine the optimal magnetization conditions allowing to maximize the torque sensitivity.

## 3. Materials and Methods

### 3.1. Investigated Material

Nickel-zinc ferrites are described by the general formula ${\mathrm{Ni}}_{x}{\mathrm{Zn}}_{1-x}{\mathrm{Fe}}_{2}O_{4}$ [30]. The crystalline structure of ferrite is composed of the two sublattices with antiparallel orientation of the non-equal magnetic moments. Thus, they exhibit the ferrimagnetic properties [26]. This leads to the generally lower saturation magnetization than in the case of ferromagnetic Fe alloys. However, due to their high electrical resistivity (10–10^7^ Ωm for Ni-Zn ferrite [30]), they are utilized in the high-frequency applications, where the power loss of Fe alloys is unacceptable due to the increasing eddy current loss. The stoichiometric ratio of Ni to Zn is crucial for the magnetic and magnetoelastic properties of Ni-Zn ferrite. Greater Zn content favors the increase in magnetic permeability. The coercive field is also lower than in the pure ${\mathrm{NiFe}}_{2}O_{4}$ [36]. On the other hand, greater Zn addition decreases the Curie temperature of the material [37], which affects the temperature range of operation. Greater Ni content leads to an increase in the saturation magnetostriction coefficient [38], which is favorable from the point of view of magnetoelastic properties.

The subject of investigation was Ni-Zn ferrite of chemical composition ${\mathrm{Ni}}_{0.3}{\mathrm{Zn}}_{0.7}{\mathrm{Fe}}_{2}O_{4}$ and characterized by average bulk density 5.1 Mg/m^3^. As the performed experiment was the first investigation of the magnetoelastic properties of Ni-Zn ferrite under torque operation, the typical chemical composition was selected as a representative of the considered group of soft ferrites. The Ni to Zn ratio results in the relatively high saturation magnetization and saturation flux density, while the coercive field is maintained at a relatively low level. The Ni content provides a significant magnetostriction coefficient favorable for intended application. Previous study of the axial stress effect on the magnetic properties of this material indicated considerable magnetoelastic sensitivity [25], which may lead to the significant torque sensitivity as well. The basic magnetic properties of the material, measured in the magnetizing field of amplitude 100 A/m at 1 Hz frequency, are summarized in Table 1. The distinctive value of the saturation magnetostriction $\lambda_{s}$ indicates favorable magnetoelastic properties.

The investigated material was prepared according to the procedure described in [38], which is commonly utilized in the ceramic industry. The basic powders of NiO, ZnO and ${\mathrm{Fe}}_{2}O_{3}$ were mixed and heated in order to initialize chemical reactions:

(11) $${\mathrm{Fe}}_{2}O_{3}+\mathrm{NiO}⟶{\mathrm{NiFe}}_{2}O_{4}$$

(12) $${\mathrm{Fe}}_{2}O_{3}+\mathrm{ZnO}⟶{\mathrm{ZnFe}}_{2}O_{4}$$

The resulting oxides were mixed in relevant proportions and wet-milled. The dried powder was prefired at a temperature of 1100 °C in order to initialize forming of the ferrite. Then, the powder was milled and mixed again. The obtained mixture was extruded into the toroidal form and pressed. Finally, the product was sintered at the temperature 1400 °C for 4 h. As a final product, the toroidal ferrite core was obtained.

Geometrical dimensions of the core and parameters of the magnetic circuit are presented in Table 2. Obtained geometry allowed to integrate the core with the mechanical setup for torque application. The core was equipped with a set of two coils in order to allow measurement of the magnetic properties: the magnetizing coil composed of 5 turns and the sensing coil composed of 25 turns.

### 3.2. Measurement Methodology

**Figure 3:**
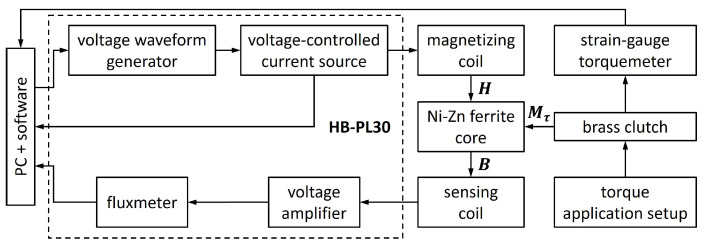
Schematic block diagram of the measurement system: *H*—magnetizing field, *B*—magnetic flux density, $M_{\tau }$—torque.

A schematic block diagram of the measurement system utilized in the performed experiment is presented in Figure 3. The system was composed of the automated hysteresisgraph and the mechanical setup for torque application and measurement. Therefore, both magnetizing field *H* and torque $M_{\tau }$ were acting on the investigated Ni-Zn ferrite core. The measured magnetic flux density *B* was varying with changes in both input quantities.

The utilized automated hysteresisgraph system was HB-PL30 developed at the Warsaw University of Technology (Warsaw, Poland). The magnetization block is composed of the voltage waveform generator and the voltage-controlled current source providing current, which flows through the magnetizing coil and produces the magnetizing field *H* according to Ampère’s law. The HB-PL30 system is capable of generating a sinusoidal or triangle magnetizing waveform of frequency from 0.1 Hz to 1 kHz and amplitude from 0.03 A to 2 A. The magnetic flux density *B* is measured with the built-in fluxmeter on the basis of the amplified voltage induced in the sensing coil. The estimated relative uncertainty of the magnetizing field and flux density measurement is about 1.5%. The proposed hysteresisgraph-based methodology of the magnetic quantities measurement seems to be the most convenient for the performed experiment due to the requirement of integration with a mechanical setup for torque generation and measurement. The application of other, more sensitive methods, like the vibrating sample magnetometry [39] or the magnetic force microscopy [40], would be less convenient.

**Figure 4:**
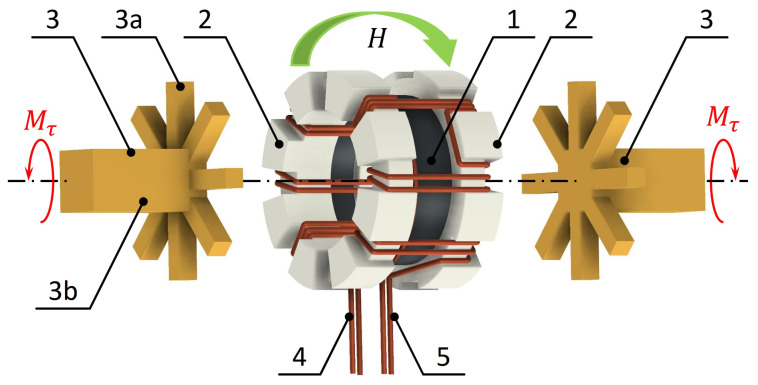
Application of the axial torque $M_{\tau }$ to the toroidal core magnetized along perimeter with the field *H*: 1—Ni-Zn ferrite core, 2—epoxy resin mold, 3—non-magnetic brass clutch (3a—beam, 3b—stem), 4—magnetizing coil, 5—sensing coil.

The methodology of torque application is similar to the one developed for amorphous toroidal cores, which was previously discussed in [17,31]. The model of the core with the torque transmitting setup is presented in Figure 4. On the base planes of the Ni-Zn ferrite toroidal core (1), the epoxy resin molds (2) were made. Each mold was formed with the set of radial grooves on the outer plane, which were used for accommodating the magnetizing (4) and sensing (5) coils. The magnetizing coil (4) provided the magnetizing field *H* acting perpendicularly to the core axis. Applied torque $M_{\tau }$ was transmitted via the non-magnetic brass clutches (3). Radial beams (3a) of the clutch (3) were matching radial grooves of the molds (2), providing the connection for torque transmission. Positions of the elements of the torque application setup were horizontally adjusted, so that the clutches were not pressing the coils. The stem (3b) of one clutch was jointed with the stationary strain-gauge torquemeter ZEPWN CL-22 (Marki, Poland) with a measurement range of 10 Nm and accuracy of 0.1%. The other clutch was jointed to the rotating shaft of the torque application setup. The torque was generated with weights placed on the pan attached with a cord to the disc rigidly mounted on the shaft. Such methodology allowed to apply torque of the intended value to the investigated core.

The investigation was performed for applied torque within the range from 0 Nm to 6.05 Nm with the step of 0.3 Nm. For each step, the magnetic characteristics of the Ni-Zn ferrite core were measured for 34 values of magnetizing field amplitude $H_{m}$ within the range from 2.5 A/m to 100 A/m (for $H_{m}$ up to 10 A/m, the step was 2.5 A/m, for higher fields, the step was 5 A/m). The investigated core was magnetized with the triangle waveform of 1 Hz frequency.

## 4. Results and Discussion

**Figure 5:**
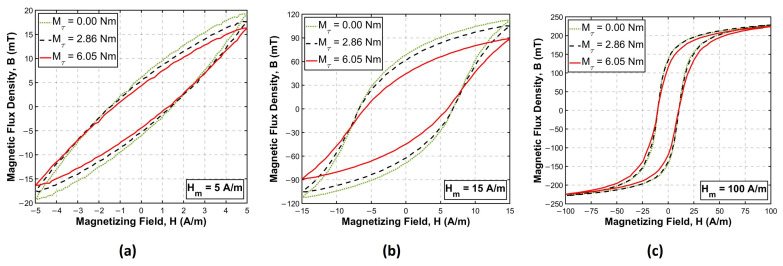
Influence of the applied torque $M_{\tau }$ on the magnetization characteristics B(H) of investigated Ni-Zn ferrite for the magnetizing field amplitude: (**a**) $H_{m}$ = 5 A/m (the Rayleigh region), (**b**) $H_{m}$ = 15 A/m (the high permeability region), (**c**) $H_{m}$ = 100 A/m (the saturation region).

The performed investigation allowed to obtain the magnetoelastic characteristics of the Ni-Zn ferrite subjected to torque. Figure 5 presents the influence of applied torque on the magnetic hysteresis loop in three different magnetization regions. Each part of the figure shows the hysteresis loops obtained for the set value of magnetizing field amplitude $H_{m}$ at three values of applied torque $M_{\tau }$, which were 0.00 Nm (unloaded), 2.86 Nm (midrange value) and 6.05 Nm (maximum value).

The hysteresis loops presented in Figure 5a were measured at $H_{m}$ = 5 A/m corresponding to the so-called Rayleigh region, where the magnetization process can be approximated with the second-degree polynomial (the Rayleigh law of magnetization) [41,42,43,44]. The obtained loops exhibit a lenticular shape characteristic for the low magnetizing fields region. Applied torque $M_{\tau }$ affects mostly the magnetic flux related parameters: the maximum magnetic flux density $B_{m}$ and the magnetic remanence $B_{r}$. The coercive field $H_{c}$ changes only for higher torque. As expected, $B_{m}$ is decreasing with the applied torque due to the growing share of magnetoelastic anisotropy in the total energy density of the material. Such behavior is favorable from the point of view of the intended torque sensing application. The observed change in $B_{m}$ allows to assess the value of torque acting on the core. However, it should be noted that for other, more typical applications of ferrite (like transformers or chokes), any change in a magnetic state under torque might be an undesirable effect, influencing the magnetic characteristics of the operating core.

Figure 5b presents the hysteresis loops measured for $H_{m}$ = 15 A/m, corresponding to the maximum relative magnetic permeability of the material. The character of the observed changes is similar to the case of the Rayleigh region (Figure 5a); however, the decrease in $B_{m}$ seems to be less linear. The greatest change in parameters occurs in the higher range of applied torque. The Zeeman energy density $w_{H}$ is stronger here than in the Rayleigh region, so for lower torque, it dominates over the magnetoelastic anisotropy $w_{\sigma }$ in the total energy density expressed with Equation (10). Therefore, for initial torque values, characteristics are shaped mostly by the magnetizing field, and observed changes are relatively low. However, with the increasing torque, the share of magnetoelastic anisotropy $w_{\sigma }$ in (10) is increasing, giving rise to more distinctive changes of $B_{m}$ and $B_{r}$ observed for higher torques.

The last set of presented loops (Figure 5c) was acquired at the field $H_{m}$ = 100 A/m, corresponding to the magnetic saturation region. The influence of torque on the magnetic parameters is relatively low. The hysteresis loops for $M_{\tau }$ = 0.00 Nm and $M_{\tau }$ = 2.86 Nm are almost identical. The maximum torque $M_{\tau }$ = 6.05 Nm affects mostly the magnetic remanence $B_{r}$, while its influence on the maximum magnetic flux density $B_{m}$ and coercive field $H_{c}$ is insignificant. Such characteristics are the result of Zeeman energy density $w_{H}$ supplied with the magnetizing field being the dominant component in Equation (10). Therefore, even for maximum torque, the magnetization vectors of magnetic domains remain oriented along the magnetizing field *H* direction rather than the anisotropy axis created by the shear stress.

**Figure 6:**
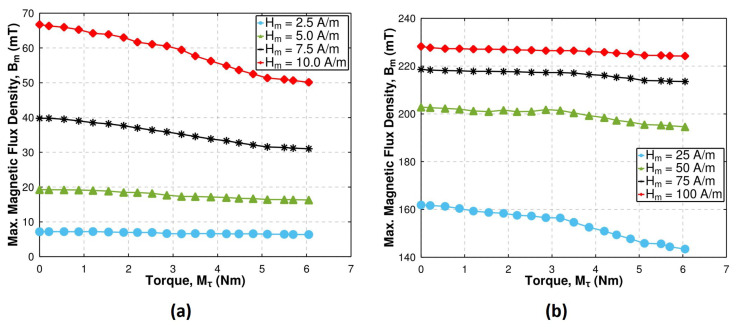
The selected magnetoelastic characteristics $B_{m}{\left(M_{\tau }\right)}_{H_{m}}$ of investigated Ni-Zn ferrite: (**a**) for lower magnetizing field amplitudes *H_m_* ≤ 10 A/m, (**b**) for higher magnetizing field amplitudes $H_{m}>$ 10 A/m: $H_{m}$ = 2.5–7.5 A/m—the Rayleigh region, $H_{m}$ = 10–25 A/m—the high permeability region, $H_{m}$ = 50–100 A/m—the saturation region.

From the point of view of the potential torque sensing application, the magnetoelastic characteristics $B_{m}{\left(M_{\tau }\right)}_{H_{m}}$ seem to be the most interesting. Such characteristics, obtained for the investigated Ni-Zn ferrite, are presented in Figure 6.

The selected characteristics shown in Figure 6a were obtained for lower magnetizing fields, mostly from the Rayleigh region and partially the high permeability region. It can be observed that the most distinctive change in $B_{m}$ under applied torque occurs for $H_{m}$ = 10 A/m, which is located at the transition between the Rayleigh region (hysteresis loop still partially exhibits lenticular shape) and the high permeability region. As it can be expected, the maximum flux density $B_{m}$ decreases with the applied torque $M_{\tau }$. The term in Equation (10), corresponding to the magnetoelastic anisotropy $w_{\sigma }$, is increasing with $M_{\tau }$, which leads to a change in the orientation of the magnetization vectors of magnetic domains, ordering them rather along the anisotropy axis than the magnetizing field direction.

Figure 6b presents the selected $B_{m}{\left(M_{\tau }\right)}_{H_{m}}$ characteristics for higher magnetizing fields. Again, the decrease in $B_{m}$ with torque $M_{\tau }$ can be observed, albeit the scale of changes is much lower, especially compared to the $B_{m}$ value in the unloaded state $M_{\tau }$ = 0 Nm. The Zeeman energy density $w_{H}$, much stronger for these fields, mostly governs the ordering of the magnetization vectors of magnetic domains, which tend to orient along the magnetizing field direction. Only for high torque, when the magnetoelastic anisotropy $w_{\sigma }$ grows stronger, the influence of torque $M_{\tau }$ on the maximum flux density $B_{m}$ is possible to observe.

On the basis of the presented characteristics, the parameters describing signal changes were determined at subsequent values of the magnetizing field amplitude $H_{m}$. The raw change in the maximum magnetic flux density $\Delta B_{m}$ was calculated as the difference between the maximum flux density in the unloaded state $B_{m}\left(M_{\tau }^{0}\right)$ and under the maximum torque $B_{m}\left(M_{\tau }^{\max}\right)$:

(13) $$\Delta B_{m}=B_{m}\left(M_{\tau }^{0}\right)-B_{m}\left(M_{\tau }^{\max}\right)$$

In terms of the magnetoelastic sensitivity, the relative change $\delta B_{m}$ is more interesting, which is calculated as

(14) $$\delta B_{m}=\frac{\Delta B_{m}}{B_{m}\left(M_{\tau }^{0}\right)}=\frac{B_{m}\left(M_{\tau }^{0}\right)-B_{m}\left(M_{\tau }^{\max}\right)}{B_{m}\left(M_{\tau }^{0}\right)}$$

Finally, the magnetoelastic torque sensitivity, $S_{M}$ expressed in 1/Nm, was calculated as

(15) $$S_{M}=\frac{\delta B_{m}}{\Delta M_{\tau }}=\frac{B_{m}\left(M_{\tau }^{0}\right)-B_{m}\left(M_{\tau }^{\max}\right)}{B_{m}\left(M_{\tau }^{0}\right)\Delta M_{\tau }}$$

where $\Delta M_{\tau }$ is the change in the applied torque throughout the entire range ($\Delta M_{\tau }$ = 6.05 Nm in performed experiment). The calculated values for selected amplitudes of the magnetizing field are summarized in Table 3. Initially, for the lower magnetizing fields up to $H_{m}$ = 20 A/m, the raw change $\Delta B_{m}$ increases up to 21.77 mT, which is the maximum value obtained. Then, the decrease in $\Delta B_{m}$ occurs with further increases in the field, where the Zeeman energy density $w_{H}$ has the dominant role in shaping the magnetic state of the material. The relative change $\delta B_{m}$ also initially increases with $H_{m}$, reaching 24.86% at $H_{m}$ = 10 A/m. The decrease in $\delta B_{m}$ starts at lower fields, as the reference value of $B_{m}\left(M_{\tau }^{0}\right)$ is rapidly increasing in the high permeability region. The obtained values of $\delta B_{m}$ at the near saturation fields are insignificant.

**Figure 7:**
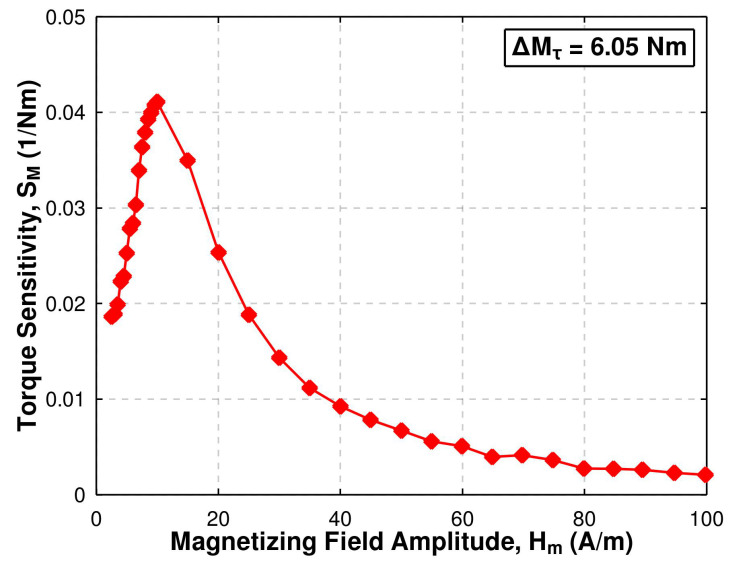
Dependence of the magnetoelastic torque sensitivity $S_{M}$ of the investigated Ni-Zn ferrite on the magnetizing field amplitude $H_{m}$ for $\Delta M_{\tau }$ = 6.05 Nm.

The dependence between the magnetoelastic torque sensitivity $S_{M}$ (15) and magnetizing field amplitude $H_{m}$ for all considered $H_{m}$ values is presented in Figure 7. Initially, the sensitivity is relatively low and begins to increase with the $H_{m}$ value. Such behavior originates from the twofold symmetry of the magnetoelastic anisotropy $w_{\sigma }$, as mentioned in Section 2. As there are two equally probable energy minima for $w_{\sigma }$ in (10), the torsion alone is not able to magnetize the material. Thus, if the magnetizing field is not strong enough to significantly influence the magnetic domains ordering, even for relatively low $M_{\tau }$, the magnetization vectors are mostly oriented along two antiparallel directions determined by the anisotropy axis at $\frac{\pi }{4}$ angle in relation to the magnetizing field. Any further increase in $M_{\tau }$ is not introducing significant change in the $B_{m}$ value, and the sensitivity is low. So, from the point of view of the sensitivity $S_{M}$, the magnetizing field has to be strong enough to introduce ordering of the magnetic domains, which then can be influenced by the shear stress resulting from the applied torque. It has to be noted that for a crystalline material, the field has to be also strong enough to overcome the magnetocrystalline anisotropy, which was not considered in the simplified approach taken in Section 2. On the other hand, the magnetizing field has to be weak enough to avoid dominance of the Zeeman energy density $w_{H}$ over the magnetoelastic anisotropy $w_{\sigma }$. This conditions for the investigated material are best fulfilled at the magnetizing field amplitude $H_{m}$ = 10 A/m, where the sensitivity $S_{M}$ reaches a maximum value of 0.041 1/Nm.

As it was mentioned in Section 1, mostly the Fe-based amorphous alloys were considered so far in terms of the torque sensing by the means of axially twisted toroidal magnetic core. In work [17], two of these materials were investigated: Fe-based alloy ${\mathrm{Fe}}_{78}B_{13}{\mathrm{Si}}_{9}$ and Fe-Ni-based alloy ${\mathrm{Fe}}_{40}{\mathrm{Ni}}_{38}{\mathrm{Mo}}_{4}B_{18}$. Both were considered in the as-quenched and annealed state. On the basis of the provided data, the sensitivity $S_{M}$ of these materials can be determined, allowing comparison with the investigated Ni-Zn ferrite. It should be noted that both alloys in [17] were characterized in the magnetizing field of amplitude $H_{m}$ = 1.5$H_{c}$. The corresponding field for the investigated ferrite would be $H_{m}$ = 15 A/m ($H_{c}$ is 10.2 A/m—Table 2), where sensitivity is 0.035 1/Nm. For the as-quenched Fe-based and Fe-Ni-based alloys, the torque sensitivity is 0.013 1/Nm and 0.001 1/Nm, respectively [17]. It can be observed that the investigated ferrite distinctively exceeds as-quenched amorphous alloys, while it does not require any additional processing to achieve such sensitivity. Only after the additional thermal annealing (1 h long [17]), amorphous alloys are able to exhibit significantly higher sensitivity: $S_{M}$ = 0.113 1/Nm for Fe-based and $S_{M}$ = 0.170 1/Nm for Fe-Ni-based alloy [17]. Therefore, in terms of the torque sensitivity, the investigated Ni-Zn ferrite can be ranked between as-quenched and annealed amorphous alloys.

## 5. Conclusions

The influence of torque on the magnetic properties of Ni-Zn ferrite was investigated for the first time. The methodology of torque application to the toroidal magnetic core, first developed for the cores coiled of amorphous ribbons, was successfully adopted for the bulk ferrite core.

The magnetoelastic characteristics of the investigated Ni-Zn ferrite obtained under torque operation are consistent with the theoretical description. The decrease in the maximum magnetic flux density occurs for the increasing torque. The scale of the observed changes is dependent on ratio of the Zeeman energy density supplied with the magnetizing field and the magnetoelastic anisotropy energy density supplied with the shear stress generated by applied torque.

As it was expected, the maximum magnetoelastic torque sensitivity is obtained for lower magnetizing field amplitude located on the verge of the Rayleigh region and the high permeability region. Sensitivity in the saturation region is significantly lower. Therefore, lower fields of amplitude about the coercive field of the material seems to be optimal for achieving the highest sensitivity. Comparing the obtained torque sensitivity with values previously reported for the Fe-based amorphous alloys, the Ni-Zn ferrite is distinctively more sensitive to the torque than as-quenched amorphous alloys. Only thermal annealing, which is the additional post-production process, allows Fe-based amorphous alloys to achieve the torque sensitivity exceeding the investigated ferrite. The high sensitivity and monotonic characteristics of the magnetic flux density changes under torque operation indicate that the investigated Ni-Zn ferrite is favorable for the intended application and can be utilized in the magnetoelastic static torque sensor. Therefore, it can be applied in the torque sensing applications required in mechanical engineering or civil engineering (evaluation of the structural elements exposed to torsion). However, it has to be noted that the measured magnetoelastic characteristics exhibit a certain non-linearity.

Further research should be concentrated on the investigation of influence of the Ni to Zn ratio and the saturation magnetostriction on the torque sensitivity. Due to the fact that ferrite properties are strongly dependent on the density and grain size, the influence of these parameters on the magnetoelastic characteristics under torque operation should be the subject of future study as well. In case of the crystalline ferrite, the role of the magnetocrystalline anisotropy in the magnetoelastic effect should also be concerned in further investigations.

## Figures and Tables

**Table 1:** Basic magnetic properties of ${\mathrm{Ni}}_{0.3}{\mathrm{Zn}}_{0.7}{\mathrm{Fe}}_{2}O_{4}$ ferrite measured in unloaded state at 100 A/m amplitude of magnetizing field.

Parameter	Unit	Value
Saturation flux density $B_{s}$	mT	230
Magnetic remanence $B_{r}$	mT	140
Coercive field $H_{c}$	A/m	10.2
Initial magnetic permeability $\mu_{i}$	–	280
Saturation magnetostriction $\lambda_{s}$	μm/m	−1.5

**Table 2:** Geometrical parameters of the investigated Ni-Zn ferrite core.

Parameter	Unit	Value
Outer diameter *D*	mm	26
Inner diameter *d*	mm	16
Thickness *h*	mm	15
Magnetic flux effective path $l_{e}$	mm	66
Effective cross-sectional area $S_{e}$	mm^2^	75

**Table 3:** The maximum magnetic flux density $B_{m}$ changes ($\Delta B_{m}$—raw change, $\delta B_{m}$—relative change) and the magnetoelastic torque sensitivity $S_{M}$ of the investigated Ni-Zn ferrite at the selected magnetizing field amplitudes $H_{m}$ for $\Delta M_{\tau }$ = 6.05 Nm.

$H_{m}$ (A/m)	$\Delta B_{m}$ (mT)	$\delta B_{m}$ (%)	$S_{M}$ (1/Nm)
2.5	0.81	11.27	0.0186
5.0	2.94	15.29	0.0253
7.5	8.75	22.00	0.0364
10.0	16.59	24.86	0.0411
20.0	21.77	15.33	0.0253
30.0	15.21	8.67	0.0143
40.0	10.73	5.59	0.0092
60.0	6.46	3.07	0.0051
80.0	3.65	1.66	0.0027
100.0	2.86	1.26	0.0021

## Data Availability

The original contributions presented in the study are included in the article; further inquiries can be directed to the corresponding author.
